# Case Report: Isolated facial and trigeminal nerve palsy without ataxia in anti-GQ1b antibody syndrome secondary to *Mycoplasma pneumonia*

**DOI:** 10.3389/fimmu.2022.1062567

**Published:** 2022-12-14

**Authors:** Shuwen Deng, Lihong Yin, Wei Lu, Song Ouyang, Weifan Yin

**Affiliations:** ^1^ Department of Health Management, The Third Xiangya Hospital, Central South University, Changsha, China; ^2^ Department of Neurology, The Second Xiangya Hospital, Central South University, Changsha, China; ^3^ Department of Neurology, The Affiliated Changsha Hospital of Xiangya School of Medicine, Central South University, Changsha, China

**Keywords:** Guillain-Barré syndrome, anti-GQ1b antibody syndrome, *Mycoplasma pneumoniae*, facial nerve, trigeminal nerve

## Abstract

The presence of anti-GQ1b antibodies in serum or cerebrospinal fluid is a diagnostic indicator of the Miller–Fisher variant of Guillain–Barré syndrome (GBS), whereas anti-GQ1b antibody syndrome is rarely presented as acute bilateral pain in the cheeks and masticatory muscle fatigue without ophthalmoplegia, ataxia, or limb weakness. Here, we report a case of a female patient diagnosed with GBS characterized only by the involvement of the facial and trigeminal nerves who was positive for serum anti-GQ1b antibodies secondary to *Mycoplasma pneumoniae* infection. The patient was treated with macrolide antibiotics and neurotrophic drugs, and her symptoms were significantly alleviated after 1 month. This case indicates a new clinical presentation of GBS and anti-GQ1b antibody syndrome with a differential diagnosis of multiple cranial nerve damage of which neurological physicians should be aware. Positive anti-GQ1b antibodies secondary to infection were observed in this case, and antibiotic treatment resulted in a favorable prognosis. The specific underlying mechanism requires further investigation.

## Introduction

GQ1b gangliosides are expressed in the extraocular neuromuscular junctions, extraocular muscle spindles, and paranodal regions of the extraocular cranial nerves (1, 2). Anti-GQ1b antibodies targeted to these regions develop in response to similar antigens from invading pathogens, such as *Campylobacter jejuni*, Cytomegalovirus, Epstein–Barr virus, or *Mycoplasma pneumoniae* (3). Anti-GQ1b antibody syndrome has overlapping clinical manifestations, including Miller–Fisher syndrome (MFS), Guillain-Barré syndrome (GBS) with ophthalmoplegia, Bickerstaff’s brain stem encephalitis, and acute ophthalmoparesis without ataxia (4).

Isolated cranial nerve damage without ataxia reported previously includes cranial nerves III, IV, VI, VII, VIII (5), and IX (6, 7); however, it is less reported in patients with anti-GQ1b antibodies in general. Herein, we report a case of a female patient diagnosed with GBS characterized only by the involvement of the facial and trigeminal nerves, who was positive for serum anti-GQ1b antibody secondary to *M. pneumoniae* infection. Diagnosis was challenging due to the atypical presentation, which supplemented the further understanding of anti-GQ1b antibody syndrome. The patient made a full recovery without the use of immunotherapy. Patients with positive anti-GQ1b antibodies do not present the full complement of MFS, which has been reported previously (3, 8). However, involvement of the facial and trigeminal nerves presenting as facial pain and limited mouth opening has not been previously reported. This report further expands the phenotypic spectrum of anti-GQ1b syndrome. The patient provided written informed consent for the publication of this report.

## Case description

A 14-year-old girl was admitted to our department because of mouth opening limitation and facial pain for 3 days. She developed a cough after a cold, accompanied by white sticky sputum. The symptoms gradually aggravated, and on day 6, she was diagnosed with pulmonary infection in the local county hospital and treated with fosfomycin sodium and ribavirin. During the pneumonia treatment, she experienced a sudden onset of mouth opening limitation and complained of bilateral pain in the cheeks with facial paralysis. Magnetic resonance image (MRI) scan of the head and electromyogram were normal. Treatment with fosfomycin sodium and ribavirin lasted for 6 days, alleviating the cough while the intracranial nerve dysfunction was more severe. She was admitted to our department 13 days after the onset of the disease. The patient had no previous medical history, including fever, tetanus, and botulism infection, and her family history was similar.

**Figure 1:**
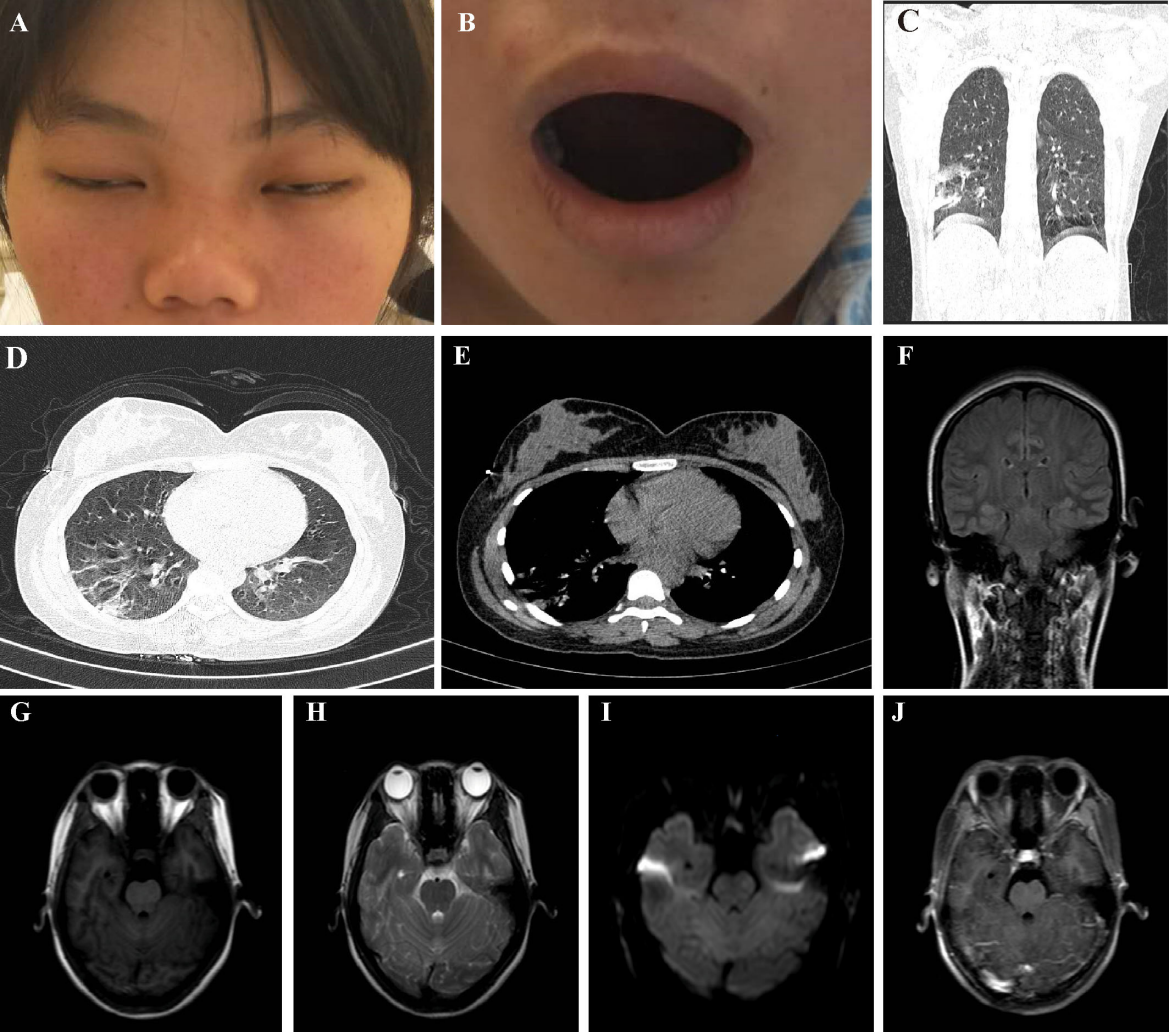
Clinical examination reveals dysfunction of facial nerve and the motor branch of the trigeminal nerve. **(A)** Bilateral facial weakness and Bell’s sign, **(B)** Limited mouth opening (tried the most effort to open her mouth); **(C-E)**. Multiple lesions in the lower lobe of the right lung; **(F-J)**. Head magnetic resonance imaging was normal.

Upon admission to our hospital, physical examinations showed dysfunction of the motor branch of the facial and trigeminal nerves (**Figures 1A, B**). The bilateral temporal and masseter muscles presented with tenderness but no atrophy, and the patient was unable to bite or open her mouth. There was no mandibular deviation, and the bilateral corneal reflex was present. The patient was unable to wrinkle her brows, bilateral forehead wrinkles disappeared, and the orbicularis oculi muscle was weakened. The patient presented shallow bilateral nasolabial muscles and an inability to show the teeth, gills drums, or whistle. The rest of the physical examination was unremarkable. She was afebrile and systemically well with no evidence of meningismus. Routine blood biochemical results were normal. Laboratory tests for infectious agents were positive for antibodies against *M. pneumoniae* (1:160), whereas cytomegalovirus, Epstein–Barr, herpes simplex virus (HSV), rubella, coxsackie, hepatitis B and C viruses, HIV, *Toxoplasma gondii*, and *Borrelia burgdorferi* were all negative. An extensive immunological investigation included autoimmune markers (ANA, APCA, ASMA, P-ANCA, C-ANCA, anti-DNA, and RA), which were normal. Cerebrospinal fluid (CSF) showed albuminocytological dissociation (pressure 140 mm H_2_O, the liquid was colorless and transparent, white cell concentration 2 ×10^6^/L, protein concentration 1338.8 mg/L, and normal glucose). CSF culture and PCR for rubella, *Borrelia*, morbilli, varicella-zoster virus, HSV type 1, and HSV type 2 were negative. The CSF was negative for ganglioside antibody, but the serum was positive for GQ1b IgG antibody.

**Figure 2:**
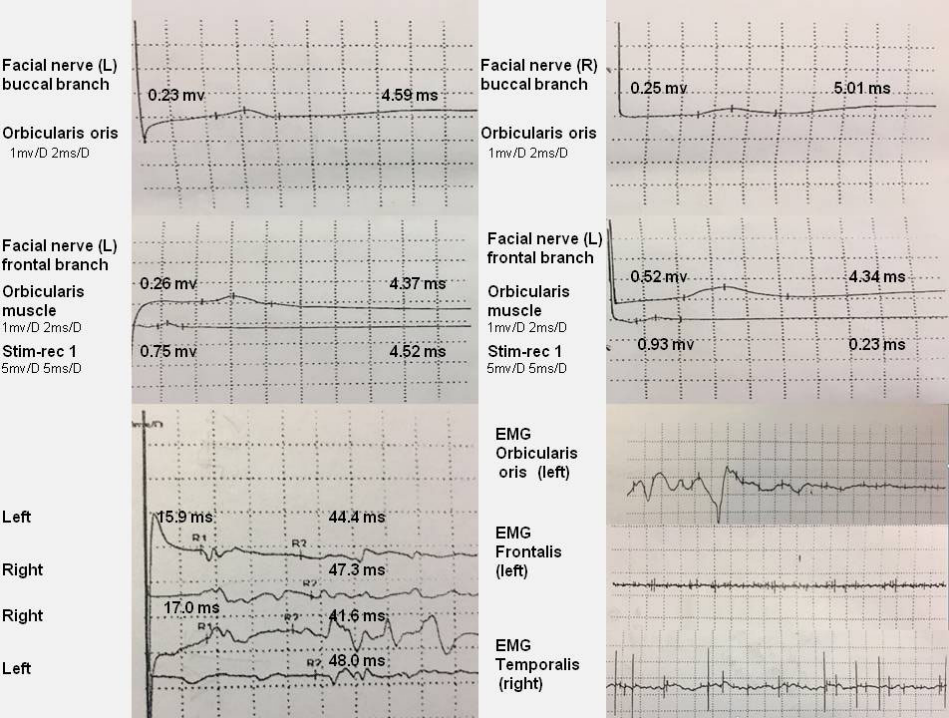
EMG reveals that the facial nerve has obvious demyelination and axonal loss. Motor nerve conduction of the bilateral facial nerve (temporal, zygomatic, and buccal branches) is prolonged, and amplitude potentials are decreased. EMG of the bilateral buccinator and orbicularis muscle and the right musculus dormitator show reduced recruitment of motor units with obvious spontaneous activity. Blink reflex R1 and R2 latency is prolonged with a discrete waveform.

A lung CT scan revealed multiple lesions in the lower lobe of the right lung, suggesting lung infection (**Figures 1C–E**). Combining these results with laboratory tests, the patient was diagnosed with *M. pneumoniae* infection. An X-ray of the mandibular joint and head MRI were normal (**Figures 1F–J**). Nerve conduction velocity and electromyography (EMG) were performed on day 15. Motor nerve conduction of the bilateral facial nerves (temporal, zygomatic, and buccal branches) were prolonged and accompanied by potential amplitude decreases. Needle EMG of the bilateral buccinator and orbicularis muscles and the right musculus dormitator showed reduced recruitment of motor units with obvious spontaneous activity, indicating demyelination and axonal loss. Blink reflex R1 and R2 latency was prolonged with a discrete waveform. The examination revealed that the bilateral blink reflex and facial nerve presented with remarkable demyelination and axonal loss (**Figure 2**). These findings, in combination with the clinical history, highly suggested a GBS variant with facial nerve dysfunction.

Given the physical exam, it was not important to diagnose viral, traumatic, and neoplastic encephalitis. The patient was diagnosed with a GBS variant involving the fifth and eighth cranial nerves, which may be related to anti-GQ1b antibodies after *M. pneumoniae* infection. In addition to the albuminocytologic dissociation in the CSF and anti-GQ1b antibodies in the serum, the findings of *M. pneumoniae* infection history, clinical manifestation, and EMG showed that the bilateral blink reflex and facial nerve presented with notable demyelination and axonal loss, all supporting a GBS diagnosis. Considering that nervous dysfunction does not involve breath and the medical economic burden, the patient was only treated with macrolide antibiotics and neurotrophic drugs. The cough and expectoration were much alleviated, and the bilateral buccal muscle pain and mouth opening restriction were also significantly improved after 1 month of treatment. At the third month of follow-up, the patient’s symptoms of facial palsy completely resolved.

## Discussion

To diagnose this case, the pathological changes in the bilateral masticator and facial nucleus were determined without involving other brain nuclei groups and pyramidal or extrapyramidal systems. The lesion involving only the bilateral trigeminal nerve motor branch and facial nerve, excluding the masticator and facial nucleus, was considered. The examination of brain MRI and EMG results demonstrated a peripheral lesion of the bilateral trigeminal nerve motor branch and facial nerve. To make an etiological diagnosis, tetanus was excluded based on the lack of exposure to tetanus toxin or *Clostridium tetani*. However, the patient was diagnosed with pneumonia before the neurological deficit appeared, subsequently anti-GQ1b and anti-*M. pneumoniae* IgG were detected in serum, and albuminocytologic dissociation was observed in the CSF. Therefore, the etiologic diagnosis was focused on inflammation and an autoimmune reaction, indicating a diagnosis of anti-GQ1b antibody syndrome with an MFS variant. A diagnosis of anti-GQ1b antibody syndrome with MFS depends on the clinical manifestation of bilateral ophthalmoplegia, decreased reflexes, and ataxia together with the absence of limb weakness and CNS involvement (9). Albuminocytological dissociation in the CSF or electrodiagnostic evidence of neuropathy are considered supplementary to support the diagnosis (9).

The patient complained mainly of facial pain and limited mouth opening, which is a new type of GBS variant that has not been reported before. The patient was first misdiagnosed as having mandibular joint disorder. However, examination of temporomandibular joint and brain MRI were conducted repeatedly and were normal, suggesting a different diagnosis. Subsequently, albuminocytologic dissociation in the CSF and *M. pneumoniae* antibodies (1:160) were identified in the serum. This case featured limited involvement of the facial and trigeminal nerves with positive serum anti-GQ1b antibody titers secondary to *M. pneumoniae* infection.

Until now, isolated cranial nerve damage without ataxia reported previously included only cranial nerves III, IV, VI, VII, VIII, and IX in anti-GQ1b antibody syndrome (**Table 1**). Respiratory infections more frequently precede the neurological onset of patients that are anti-GQ1b positive, whereas *C. jejuni* infection is a preceding infection in a smaller proportion of patients. In a clinical study including 213 patients with GBS, 58% of pediatric patients with GBS had *M. pneumoniae*-specific IgG antibodies in the serum compared with 19% of healthy children, whereas these percentages were 34% and 12%, respectively, in adults (17). The most common gangliosides antibody secondary to *M. pneumoniae* infection is anti-GalC antibodies (18). *M. pneumoniae* infection is the most important inducement of GBS (19). However, generation of anti-GQ1b antibodies secondary to *M. pneumoniae* infection is not common (19). A possible relationship between anti-GQ1b antibodies and *M. pneumoniae* infection was intriguing. Potential mechanisms involved in the generation of anti-GQ1b antibodies secondary to *M. pneumoniae* infection are presented as follows: (і) molecular mimicry—the *M. pneumoniae* cytoplasm contains potent immunogenic substances (e.g., glycolipids and glycoproteins) that elicit autoimmunity through molecular mimicry of various human cellular components, such as those of brain tissue (2, 20). However, the molecular mimicry between GQ1b and *M. pneumoniae* has not been confirmed. (ii) Abnormal antigen expression of GQ1b—we hypothesized that *M. pneumoniae* may enter the body and change the antigenicity of cells not expressing GQ1b, inducing the production of antibodies and T-cell reactions, resulting in antigen–antibody combinations leading to neurological manifestation.

**Table 1:** Isolated cranial nerve damage reported in anti-GQ1b antibody syndrome.

Ref	Cranial nerve	Age	Sex	Previous medical history before onset	Antibody	Immunotherapy	Recovery Period
(10)	Oculomotor nerve palsy (right)	68	Man	*Campylobacter* infection	Anti-GQ1b antibodies in serum	No	87 days
(11)	Oculomotor nerve palsy	65	Man	Injection of the COVID-19 vaccine	Anti-GQ1b antibodies in serum	Intravenous immunoglobulin (IVIG, 400 mg/kg) for 5 consecutive days	71 days
(12)	Bilateral sixth nerve palsies, a vertical gaze palsy, a nasal and hoarse voice, an impairment of swallowing and bilateral facial palsies	52	Man	Upper respiratory tract infection	Anti-GQ1b antibodies in serum	Immunoglobulin treatment (IVIG, 400 mg/kg) for 5 consecutive days	2 months
(13)	Bilateral ophthalmoplegia with right lower motor neuron facial palsy	14	Man	Fever and cough lasting for 4 days 15-d prior	Anti-GQ1b antibodies in serum	No	21 days
(14)	Asymmetrical and isolated hypoglossal nerve palsy	75	Man	Malignant lymphoma	Anti-GQ1b antibodies in serum	Chemotherapy and prednisolone (70 mg for 5 days).	Died of *Pneumocystis* pneumonia after 2 weeks of chemotherapy
(15)	Isolated optic disc edema in both eyes	75	Man	Diarrhea and myalgia two weeks before presentation	Anti-GQ1b antibodies in serum	Intravenous methylprednisolone (1 g/day) for 5 consecutive days and 500 mg per day of oral acetazolamide	2 months
(8)	Glossopharyngeal nerve and vagus nerve bilaterally	12	Man	*Mycoplasma* pneumonia	Anti-GQ1b antibodies in CSF	Intravenous immunoglobulins (1000 mg/kg/day for 2 days	6 weeks
(16)	Bilateral visual deterioration (right 0.6 and left 0.3), dysarthria and facial palsy	73	Man	Acute diarrhea	Anti-GQ1b antibodies were positive in both serum and cerebrospinal fluid	Intravenous methylprednisolone (500 mg/d) for 2 days and immunoglobulin (32.5 g/d) was administered for 5 days	20 days

**Figure 3:**
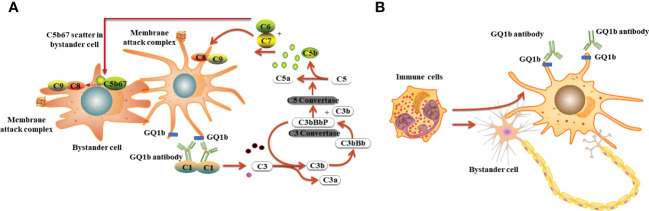
The possible mechanisms underlying anti-GQ1b antibody syndrome following *M. pneumoniae* infection. **(A)** Complement dependent cytotoxicity: antibody binding to antigen produced antigen–antibody complexes, followed by activated, soluble complement protein deposition forming a membrane-attack complex on nearby non-GQ1b expressing cells, resulting in bystander injury and causing damage to cells not expressing GQ1b; **(B)** Antibody-dependent cell-mediated cytotoxicity: antigen–antibody complex formation recruiting T cells or other immune cells to engulf damaged cells including nearby non-GQ1b expressing cells.

*M. pneumoniae*–related anti-GQ1b antibody syndrome presents as Bickerstaff brainstem encephalitis (21), MFS, GBS with ophthalmoplegia (22), or isolated bulbar involvement (8). The ophthalmoplegia and oropharyngeal symptoms frequently observed in various anti-GQ1b syndromes are due to the abundant expression of ganglioside GQ1b on the cranial nerves, including the oculomotor (III), trochlear (IV), abducens nerves (VI), glossopharyngeal (XI), and vagal nerves (XI) (23). As far as we know, no reports indicate the binding of anti-GQ1b autoantibodies to the trigeminal nerve (V). This is the first case of anti-GQ1b antibody syndrome presenting as bilateral cheek pain and masticatory muscle fatigue without ophthalmoplegia, bulbar paralysis, limb weakness, ataxia, and sensory disturbance. However, whether ganglioside GQ1b is abundant on cranial nerve V and how anti-GQ1b antibodies result in damage requires further investigation. We hypothesized that antibody binding to antigen produced antigen–antibody complexes, followed by activated, soluble complement protein deposition forming a membrane-attack complex on nearby non-GQ1b expressing cells, resulting in “bystander injury” and causing damage to cells not expressing GQ1b (24, 31). In addition, antigen–antibody complex formation may recruit T cells and phagocytic cells to engulf damaged cells, including nearby non-GQ1b expressing cells. Therefore, the bystander injury is derived from complement-dependent cytotoxicity or antibody-dependent cell-mediated cytotoxicity (24, 31) (**Figure 3**). The specific mechanism requires further investigation.

Treatment of MFS is controversial because MFS is a self-limited disease (25). Mori et al. have found that intravenous immunoglobulin or plasmapheresis therapy does not seem to influence patient outcomes though it slightly hastens the alleviation of ophthalmoplegia and ataxia (32, 26). In line with previous studies, the case reported here was treated with only macrolide antibiotics and neurotrophic drugs and had a full recovery after long-term follow-up. However, some cases suggest the possible efficacy of immunomodulating therapy in MFS, where the choice of immunotherapy is based on the severity of clinical symptoms, such as profound ataxia, severe bulbar palsy, respiratory and motor impairment, or during pregnancy (27–29). Therefore, clinicians should not treat pure MFS using immunomodulating therapy until there is enough supporting evidence (30).

## Conclusions

Patients presenting with only bilateral paralysis, facial muscle pain, and masticatory dysfunction may be assessed for a variant of GBS. Besides CSF examination, including cell count and biochemical analysis, ganglioside antibody spectrum detection and careful EMG examination should be performed. Once diagnosis of a rare type of GBS is suspected, treatment should be administered immediately. Active determination of inducing factors is indispensable for rare cases of GBS because they are both helpful for treatment and target the prevention of critical GBS presented as dyspnea. Finally, a different treatment may be administered depending on the severity of GBS. In this case, treatment with macrolide antibiotics successfully treated the pneumonia as well as the neurological deficit, without immunoregulatory treatment.

## Data availability statement

The original contributions presented in the study are included in the article/supplementary material. Further inquiries can be directed to the corresponding author.

## Ethics statement

Written informed consent was obtained from the individual(s) for the publication of any potentially identifiable images or data included in this article.

## Author contributions

SD drafted the manuscript. SO collected the data. LY and WL carried out the literature review. WY finalized the draft. All authors read and approved the final manuscript.
